# Body Roundness Index Associated With Cardiometabolic Multimorbidity and Mortality: A Multistate Model

**DOI:** 10.1002/oby.70032

**Published:** 2025-09-28

**Authors:** Xi Cai, Yicheng Liao, Xuemei Yang, Yajing Liang, Jiajia Ma, Ruiyue Liu, Xinran Wen, Wenli Yin, Shuohua Chen, Guodong Wang, Na Li, Shouling Wu, Liufu Cui

**Affiliations:** ^1^ Department of Rheumatology and Immunology Kailuan General Hospital, North China University of Science and Technology Tangshan Hebei China; ^2^ Department of Cardiology Kailuan General Hospital, North China University of Science and Technology Tangshan Hebei China; ^3^ Department of Cardiology First Affiliated Hospital of Kunming Medical University Kunming Yunnan China

**Keywords:** body roundness index, cardiometabolic disease, cardiometabolic multimorbidity, mortality, progression

## Abstract

**Objective:**

This study aimed to investigate the associations of body roundness index (BRI) with cardiometabolic disease (CMD), cardiometabolic multimorbidity (CMM), and all‐cause mortality, while evaluating its impact across different stages of CMM progression.

**Methods:**

In this prospective cohort study, 87,902 participants from the Kailuan cohort were categorized into BRI quartiles. Cox models estimated hazard ratios (HRs) and 95% CIs for the first occurrence of cardiometabolic disease (FCMD), CMM, and mortality. Multistate models assessed BRI's role across CMM progression.

**Results:**

Over a median follow‐up of 13.68 years, 21,636 participants developed FCMD, 2114 developed CMM, and 14,782 died. Elevated BRI increased risks of FCMD, CMM, and mortality in Cox models. Multistate analysis revealed differential BRI effects across CMM progression: participants in the highest versus lowest BRI quartile showed HRs of 2.08 (1.99–2.17) for healthy‐to‐FCMD transition, 1.61 (1.38–1.88) for FCMD‐to‐CMM transition, and 1.09 (1.03–1.16), 0.99 (0.89–1.10), and 0.73 (0.54–0.99) for mortality from the healthy state, FCMD, and CMM, respectively. BRI's impact varied by disease type (diabetes mellitus, myocardial infarction, stroke) and sex, with stronger associations in females.

**Conclusions:**

Our findings emphasize dynamic BRI monitoring as a biomarker for early CMM risk identification and prognostic assessment, necessitating disease‐ and sex‐specific prevention strategies.

**Study Importance**

- What is already known?
  ○ Obesity metrics like BMI inadequately distinguish central adiposity from general adiposity, limiting their utility for predicting multimorbidity.
  ○ Body roundness index (BRI) shows superior predictive value for single cardiometabolic diseases and mortality compared to BMI, but its role in cardiometabolic multimorbidity (CMM) progression remains unexplored.
- What does this study add?
  ○ Using multistate models, we demonstrate that elevated BRI differentially influences progression from health to first occurrence of cardiometabolic disease (FCMD), to CMM, then to death.
  ○ BRI's association with adverse outcomes is stronger in females. Disease‐specific analyses show BRI most strongly drives diabetes onset and myocardial infarction progression to CMM.
- How might these results change the direction of research or the focus of clinical practice?
  ○ BRI should be integrated into routine screenings as a dynamic biomarker to identify high‐risk individuals (especially normal‐BMI central obesity) and guide early interventions before FCMD onset. Sex‐specific BRI thresholds are needed for precision prevention.
  ○ The protective effect of high BRI in late‐stage CMM challenges conventional paradigms, urging research into mechanisms.

## 1 Introduction

Cardiometabolic multimorbidity (CMM) is defined as the coexistence of two or more cardiometabolic diseases (CMDs), such as diabetes mellitus (DM), coronary artery disease, and cerebrovascular accidents [1, 2]. In recent years, CMDs have emerged as a critical global health priority, accounting for more than 10 million deaths annually while their prevalence continues to rise [3, 4, 5, 6, 7, 8]. Compared to single CMDs, CMM significantly increases mortality risk and reduces life expectancy [1]. This comorbid state not only heightens individual health risks but also places a substantial burden on health care systems [9]. Therefore, identifying modifiable risk factors for both CMDs and CMM at an early stage is essential.

Obesity is a well‐established, independent risk factor for CMM and is traditionally assessed with the body mass index (BMI) [2]. However, BMI fails to distinguish central obesity—a key driver of cardiometabolic risk—from general adiposity. Individuals with a normal BMI but central obesity show higher CMD prevalence and mortality than their counterparts without central obesity at the same BMI [10]. To address this limitation, the body roundness index (BRI)—a novel anthropometric measure derived from waist circumference (WC) and height—has attracted growing attention [11]. Recent studies show that BRI outperforms other obesity metrics in predicting adverse outcomes like hyperuricemia and metabolic syndrome [12, 13] and that an elevated BRI is an independent risk factor for several individual CMDs [14, 15]. Nevertheless, evidence on its relation to CMM and on how it influences the dynamic progression of CMM remains scarce.

Therefore, using data from the Kailuan cohort study (registration number: ChiCTR‐TNRC‐11001489), we aim to investigate the associations of BRI with incident CMDs, CMM, and all‐cause mortality, while evaluating its impact across different stages of CMM progression.

## 2 Methods

### 2.1 Study Participants

The Kailuan Study, which started in 2006, is a large prospective cohort study of active and retired workers from the Kailuan Group, Tangshan, China. For this study, examinations—including measurements of height, WC, and fasting blood glucose (FBG)—were conducted biennially. Participants were included in this study based on the following inclusion criteria: (1) they participated in the 2006 health checkup; (2) their data on WC and height were available; (3) they agreed to participate in this study and signed the informed consent form. Exclusion criteria were: (1) they had a history of DM before the health checkup in 2006; (2) they had a history of myocardial infarction (MI) before the health checkup in 2006; (3) they had a history of stroke before the health checkup in 2006 (Figure S1). This study was approved by the Ethics Committee of Kailuan General Hospital (approval number: 200605) and conducted in accordance with the Declaration of Helsinki.

### 2.2 Data Collection

Anthropometric indicators, biochemical determinations, and epidemiological investigations have been described in the published papers from our team [16]. Anthropometric indicators included WC, height, weight, and blood pressure. Biochemical indices included FBG, high‐density lipoprotein cholesterol (HDL‐C), low‐density lipoprotein cholesterol (LDL‐C), serum creatinine (SCr), and high‐sensitivity C‐reactive protein (hs‐CRP). Epidemiological information covered demographic information (sex, age, income level, education level, marital status), health behavioral habits (smoking, alcohol consumption, physical activity), disease history (history of hypertension, history of DM), and medication history (use of antihypertensive and lipid‐lowering medications).

### 2.3 Relevant Definitions

Hypertension was defined as systolic blood pressure ≥ 140 mmHg and/or diastolic blood pressure ≥ 90 mmHg or as systolic blood pressure < 140 mmHg and diastolic blood pressure < 90 mmHg but with a history of definitively diagnosed hypertension or current antihypertensive therapy [17]. Physical activity was defined as exercising at least three times per week for ≥ 30 min per session. Smoking was defined as smoking on average at least one cigarette per day during the past year. Alcohol consumption was defined as a mean daily intake of about 100 mL of alcohol (with ≥ 50% alcohol content) for at least 1 year. BMI was calculated as weight in kilograms divided by height in meters squared. The estimated glomerular filtration rate (eGFR) was calculated using the Chronic Kidney Disease Epidemiology Collaborative Study formula [18].

### 2.4 Exposure Variable

BRI is calculated as 364.2–365.5 × (1 − [WC (m)/2*π*]^2^/[0.5 × height (m)]^2^)^1/2^ [11]. The 25th, 50th, and 75th percentile values of BRI were calculated and divided into quartile 1 (Q1), quartile 2 (Q2), quartile 3 (Q3), and quartile 4 (Q4) (Q1: BRI < 2.95; Q2: 2.95 ≤ BRI < 3.64; Q3: 3.64 ≤ BRI < 4.42; Q4: BRI ≥ 4.42).

### 2.5 Outcome and Follow‐Up Time

Outcome events included first occurrence of cardiometabolic disease (FCMD), CMM, and all‐cause mortality. FCMD was defined as the first occurrence of DM, MI, or stroke during follow‐up. Causes of events were recorded with the International Classification of Diseases, Tenth Revision (ICD‐10), including E10–E14 for DM, I21 for MI, and I60–I69 for stroke. DM was diagnosed as FBG ≥ 7.0 mmol/L or FBG < 7.0 mmol/L with a history of clearly diagnosed DM or current hypoglycemic therapy [19]. MI followed the World Health Organization Multinational Monitoring of Trends and Determinants in Cardiovascular Disease criteria [20]. Stroke was identified based on clinical signs and neuroimaging obtained by brain computed tomography or magnetic resonance imaging, including cerebral infarction and intracranial and subarachnoid hemorrhage [21, 22]. CMM was defined as the concurrent diagnosis of two or more conditions among DM, MI, or stroke during follow‐up, with the onset date corresponding to the diagnosis date of the second CMD. Confirmation of death was based on information from local government vital statistics offices.

The 2006 health checkup completion date served as the baseline for follow‐up. Participants were followed from enrollment until death, loss to follow‐up, or December 31, 2022, whichever came first.

### 2.6 Statistical Analysis

Normally distributed measures were expressed as mean ± standard deviation (SD) ($\bar{x}$ ± *s*), and comparisons between groups were conducted using ANOVA. Measures with skewed distribution were presented as median (P25, P75), and comparisons between groups were conducted using the nonparametric rank sum test (Kruskal–Wallis). Count data were presented as frequencies and percentages, and comparisons between groups were conducted using the *χ* ^2^ test.

The cumulative incidence of outcome across different groups was calculated using the Kaplan–Meier method, and comparisons between groups were performed using the log‐rank test. The association of BRI with outcome events was analyzed using a Cox proportional hazards regression model, taking the lowest quartile of BRI as the reference. In addition to age‐ and sex‐adjusted models, multiadjusted models were also fitted, with further adjustment for other baseline covariates, including smoking status, drinking status, physical activity, income level, education level, marital status, HDL‐C, LDL‐C, hs‐CRP, eGFR, hypertension, antihypertensive drugs, and lipid‐lowering drugs. The proportional hazards assumption for Cox models was verified using Schoenfeld residual plots, with no significant violations detected. The dose–response relationship between BRI and the risk of each outcome event was analyzed using restricted cubic spline (RCS) plots with nodes placed at the 30th, 60th, and 90th percentiles of BRI, respectively.

**FIGURE 1:**
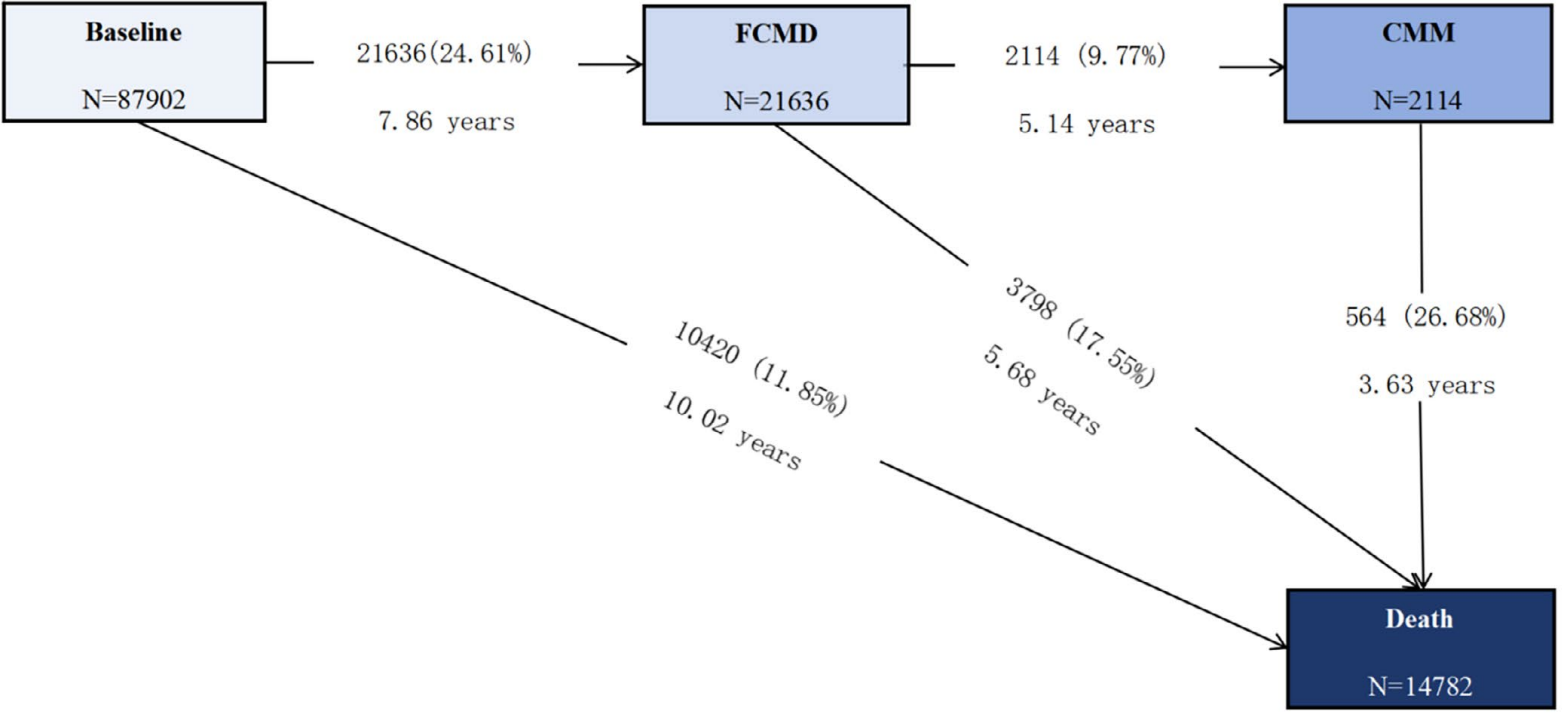
Frequency (percentage) and mean follow‐up time (years) of participants in Transition Pattern A from a baseline healthy state to first cardiometabolic disease (FCMD), cardiometabolic multimorbidity (CMM), and all‐cause mortality. [Color figure can be viewed at wileyonlinelibrary.com]

**FIGURE 2:**
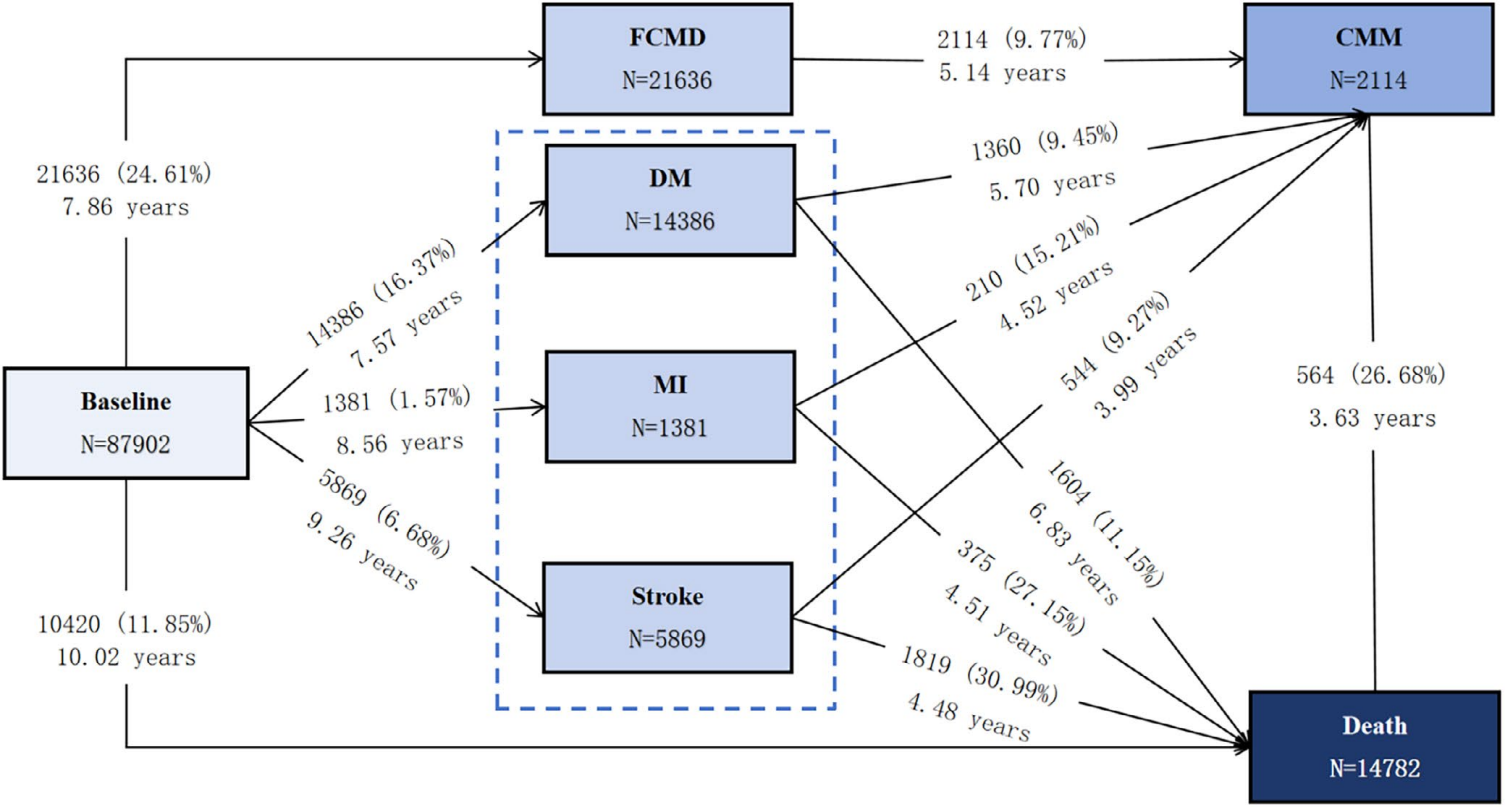
Frequency (percentage) and mean follow‐up time (years) of participants in Transition Pattern B from a baseline healthy state to one CMD, then to CMM, and subsequently to death. CMDs include diabetes mellitus (DM), myocardial infarction (MI), and stroke. [Color figure can be viewed at wileyonlinelibrary.com]

A unidirectional multistate model with Markov proportional hazards was employed to assess the impact of BRI across transitions in CMM progression and prognosis. Based on the natural course of CMM and previous studies [2], Transition Pattern A was constructed with five pathways (Figure 1): (1) baseline healthy state (free of CMDs) to FCMD, (2) baseline healthy state to death, (3) FCMD to CMM, (4) FCMD to death, and (5) CMM to death. For participants entering different states on the same date, we adopted a previously reported method [2], where the onset date of the theoretically prior disease state was set to 0.5 days before the onset date of the latter state. For example, in cases where FCMD and CMM occurred on the same day, the FCMD onset date was set as the CMM onset date minus 0.5 days. To further explore potential associations between BRI and the progression of distinct CMDs, Transition Pattern B was developed (Figure 2). In this model, based on previous studies [2], multistate pathways were expanded to three independent CMDs including DM, MI, and stroke, which comprised a total of 11 transition pathways. These pathways included transitions from the healthy state to one CMD and from individual CMDs to CMM or death.

To explore the potential modifying role of sex in the associations between BRI and outcome events, as well as its impact on CMM progression and prognosis, this study conducted sex‐stratified analyses. First, Cox proportional hazards regression models were used to analyze the associations between BRI and each outcome event (FCMD, CMM, and all‐cause mortality) separately in males and females. Second, within the multistate model of Transition Pattern A, sex‐stratified analyses were performed to further evaluate the influence of BRI on each transition pathway in different sexes.

To evaluate the robustness of the results, we further performed several sensitivity analyses, which included the following: (1) further adjusting for baseline BMI, (2) excluding events occurring in the first 2 years of follow‐up (*n* = 460), (3) excluding participants with a history of cancer (*n* = 5686), (4) using different time intervals (0.5, 1, 3, and 5 years) instead of 0.5 day for participants who entered different states on the same day, and (5) excluding participants who entered different states on the same date (*n* = 7).

Data were analyzed using SAS 9.4 (SAS Institute Inc., Cary, North Carolina) and R (version 4.4.2) statistical software. The multistate model was performed using “mstate” package in R. Two‐sided tests were performed with *p* < 0.05 indicating a statistical difference.

## 3 Results

### 3.1 Descriptive Analysis

A total of 101,510 participants underwent the 2006 health checkup of the Kailuan Study. After excluding 9493 with DM, 1082 with MI, 1617 with stroke, and 1416 with missing data on height and WC, finally a total of 87,902 participants met the inclusion criteria (Figure S1). The mean age was 50.87 ± 12.57 years, and 69,695 were male. With an increase in BRI level from Q1 to Q4, age, hs‐CRP, WC, BMI, and FBG progressively increased, whereas eGFR, HDL‐C, and height decreased. The proportions of hypertension and of individuals taking antihypertensive and lipid‐lowering drugs showed an increasing trend, whereas the proportions of those with a high school education or higher showed a decreasing trend. The differences between the groups were all statistically significant (all *p* values < 0.0001) (Table S1).

In this study with a mean follow‐up time (MFT) of 13.68 ± 4.17 years, 21,636 participants (24.61%) developed FCMD with an MFT of 7.86 years during follow‐up. Among FCMD patients, 2114 cases (9.77%) progressed to CMM with an MFT of 5.14 years. A total of 14,782 deaths were recorded during follow‐up, including 3798 FCMD patients (MFT: 5.68 years) and 564 CMM patients (MFT: 3.63 years) (Figure 1). When further dividing FCMD into three distinct CMDs, the cohort comprised 14,386 (16.37%) DM cases, 1381 (1.57%) MI cases, and 5869 (6.68%) stroke cases. Among these patients, 1360 (9.45%) with DM, 210 (15.21%) with MI, and 544 (9.27%) with stroke subsequently progressed to CMM. Additionally, 1604 (11.15%) DM patients, 375 (27.15%) MI patients, and 1819 (30.99%) stroke patients died during follow‐up (Figure 2).

### 3.2 Association of BRI With Incident FCMD, CMM, and All‐Cause Mortality

Incidence rates per 1000 person‐years rose across BRI quartiles: 10.47, 16.17, 21.17, and 27.68 for FCMD; 0.64, 1.28, 1.75, and 2.80 for CMM; and 7.46, 9.76, 11.41, and 16.08 for all‐cause mortality (Table 1). Kaplan–Meier curves showed progressively higher cumulative incidences with increasing BRI (log‐rank *p* < 0.001) (Figure S2).

**TABLE 1:** Associations of BRI with FCMD, CMM, and all‐cause mortality.

	Quartile 1	Quartile 2	Quartile 3	Quartile 4	Per SD increase
FCMD					
Case/total (incidence rate^a^)	3290/21972 (10.47)	4870/22046 (16.17)	6096/22008 (21.17)	7380/21876 (27.68)	NA
Mean survival times (years)	8.26	8.19	7.92	7.42	NA
HR (95% CI)					
Model 1	Ref.	1.44 (1.38–1.51)	1.84 (1.77–1.92)	2.36 (2.26–2.46)	1.22 (1.21–1.23)
Model 2	Ref.	1.35 (1.29–1.41)	1.69 (1.61–1.76)	2.08 (1.99–2.17)	1.19 (1.18–1.20)
CMM					
Case/total (incidence rate^a^)	215/21972 (0.64)	427/22046 (1.28)	578/(1.75)	894/21876 (2.80)	NA
Mean survival times (years)	11.55	10.83	10.93	10.86	NA
HR (95% CI)					
Model 1	Ref.	1.79 (1.52–2.11)	2.35 (2.01–2.75)	3.65 (3.14–4.24)	1.27 (1.24–1.29)
Model 2	Ref.	1.63 (1.38–1.92)	2.05 (1.75–2.40)	2.99 (2.57–3.49)	1.23 (1.20–1.26)
All‐cause mortality					
Case/total (incidence rate^a^)	2518/21972 (7.46)	3276/22046 (9.76)	3798/22008 (11.41)	5190/21876 (16.08)	NA
Mean survival times (years)	10.28	10.59	10.63	10.55	NA
HR (95% CI)					
Model 1	Ref.	1.06 (1.00–1.12)	1.09 (1.04–1.15)	1.25 (1.19–1.31)	1.09 (1.07–1.10)
Model 2	Ref.	1.02 (0.97–1.07)	1.04 (0.98–1.09)	1.14 (1.09–1.20)	1.06 (1.04–1.07)

*Note*: Model 1: adjusted for age, sex. Model 2: included variables in model 1 and further adjusted for smoking status, drinking status, physical activity, income level, education level, marital status, HDL‐C, LDL‐C, hs‐CRP, eGFR, hypertension, antihypertensive drugs, and lipid‐lowering drugs.

Abbreviations: BRI, body roundness index; CMM, cardiometabolic multimorbidity; eGFR, estimated glomerular filtration rate; FCMD, first cardiometabolic disease; HDL‐C, high‐density lipoprotein cholesterol; HR, hazard ratio; hs‐CRP, high‐sensitivity C‐reactive protein; LDL‐C, low‐density lipoprotein cholesterol.

^a^ Incidence rate per 1000 person‐years.

Cox proportional hazards regression models using BRI quartiles as the exposure and incident FCMD, CMM, or all‐cause mortality utilization separately demonstrated that Q4 had significantly elevated risks compared to Q1 for FCMD, CMM, and mortality. After adjusting for confounders, hazard ratios (HRs) for FCMD in Q2, Q3, and Q4 were 1.35 (95% CI: 1.29–1.41), 1.69 (1.61–1.76), and 2.08 (1.99–2.17), respectively. HRs for CMM were 1.63 (1.38–1.92), 2.05 (1.75–2.40), and 2.99 (2.57–3.49), and for all‐cause mortality, 1.02 (0.97–1.07), 1.04 (0.98–1.09), and 1.14 (1.09–1.20). Each 1‐SD increase in BRI was associated with HRs of 1.19 (1.18–1.20) for FCMD, 1.23 (1.20–1.26) for CMM, and 1.06 (1.04–1.07) for mortality (Table 1).

RCS analyses, after adjusting for confounders, showed an S‐shaped relationship between baseline BRI and risks of FCMD and CMM (*p* for overall association < 0.0001, *p* for nonlinear association < 0.0001), while a linear association was observed for all‐cause mortality (*p* for nonlinear association = 0.0833) (Figure S3).

### 3.3 Multistate Analyses

Using a multistate model, we further evaluated the role of BRI in transitions across CMM progression and prognosis. In Transition Pattern A, elevated BRI also significantly increased the risk of transitioning from a healthy state to FCMD, consistent with the Cox regression analyses, with Q4 exhibiting an HR of 2.08 (1.99–2.17) compared to Q1. The risk of progressing from FCMD to CMM also rose with higher BRI levels, showing an HR of 1.61 (1.38–1.88) for Q4. Conversely, among CMM patients, higher BRI was associated with a reduced risk of death (Q4 HR: 0.73, 0.54–0.99) (Table 2).

**TABLE 2:** Associations of BRI with morbidity transitions from a baseline healthy state to FCMD, CMM, and death.

	No of events (%)	HR (95% CI)				
		Quartile 1	Quartile 2	Quartile 3	Quartile 4	Per SD increase
Transition Pattern A						
Health → FCMD	21,636 (24.61%)	Ref.	1.35 (1.29–1.41)	1.69 (1.61–1.76)	2.08 (1.99–2.17)	1.19 (1.18–1.20)
Health → Death	10,420 (11.85%)	Ref.	1.00 (0.94–1.06)	1.00 (0.94–1.06)	1.09 (1.03–1.16)	1.04 (1.02–1.06)
FCMD → CMM	2114 (9.77%)	Ref.	1.30 (1.10–1.53)	1.32 (1.13–1.55)	1.61 (1.38–1.88)	1.12 (1.08–1.16)
FCMD → Death	3798 (17.55%)	Ref.	0.98 (0.87–1.10)	0.94 (0.84–1.05)	0.99 (0.89–1.10)	1.01 (0.98–1.05)
CMM → Death	564 (26.68%)	Ref.	0.82 (0.59–1.14)	0.73 (0.53–1.01)	0.73 (0.54–0.99)	0.89 (0.81–0.97)
Transition Pattern B						
Health → FCMD						
Health → DM	14,386 (16.37%)	Ref.	1.44 (1.37–1.53)	1.90 (1.81–2.01)	2.48 (2.36–2.62)	1.22 (1.21–1.23)
Health → MI	1381 (1.57%)	Ref.	1.30 (1.09–1.55)	1.61 (1.36–1.91)	1.66 (1.40–1.97)	1.13 (1.08–1.18)
Health → stroke	5869 (6.68%)	Ref.	1.21 (1.11–1.31)	1.32 (1.22–1.43)	1.47 (1.36–1.60)	1.11 (1.08–1.13)
Health → Death	10,420 (11.85%)	Ref.	1.00 (0.94–1.06)	1.00 (0.94–1.06)	1.09 (1.03–1.16)	1.04 (1.02–1.06)
FCMD → CMM						
DM → CMM	1360 (9.45%)	Ref.	1.32 (1.07–1.63)	1.25 (1.02–1.52)	1.58 (1.30–1.92)	1.09 (1.03–1.15)
MI → CMM	210 (15.21%)	Ref.	1.33 (0.77–2.27)	1.60 (0.96–2.66)	1.85 (1.12–3.05)	1.12 (1.03–1.21)
Stroke → CMM	544 (9.27%)	Ref.	1.25 (0.92–1.70)	1.40 (1.04–1.89)	1.66 (1.24–2.22)	1.17 (1.08–1.26)
FCMD → Death						
DM → Death	1604 (11.15%)	Ref.	0.95 (0.79–1.15)	1.00 (0.84–1.19)	1.18 (1.00–1.39)	1.10 (1.05–1.15)
MI → Death	375 (27.15%)	Ref.	1.03 (0.70–1.52)	1.11 (0.76–1.60)	1.19 (0.82–1.71)	1.04 (0.94–1.15)
Stroke → Death	1819 (30.99%)	Ref.	1.02 (0.87–1.20)	0.97 (0.83–1.13)	0.98 (0.84–1.13)	0.99 (0.94–1.04)
CMM → Death	564 (26.68%)	Ref.	0.82 (0.59–1.14)	0.73 (0.53–1.01)	0.73 (0.53–0.99)	0.89 (0.81–0.97)

*Note*: Model adjusted for age, sex, smoking status, drinking status, physical activity, income level, education level, marital status, HDL‐C, LDL‐C, hs‐CRP, eGFR, hypertension, antihypertensive drugs, and lipid‐lowering drugs.

Abbreviations: BRI, body roundness index; CMM, cardiometabolic multimorbidity; DM, diabetes mellitus; eGFR, estimated glomerular filtration rate; FCMD, first cardiometabolic disease; HDL‐C, high‐density lipoprotein cholesterol; HR, hazard ratio; hs‐CRP, high‐sensitivity C‐reactive protein; LDL‐C, low‐density lipoprotein cholesterol; MI, myocardial infarction.

In Transition Pattern B, analyses of transitions from a healthy status to distinct FCMD subtypes revealed that elevated BRI significantly increased risks of developing DM (Q4 HR: 2.48, 2.36–2.62), MI (Q4 HR: 1.66, 1.40–1.97), and stroke (Q4 HR: 1.47, 1.36–1.60). Among patients with FCMD, those with MI in the highest BRI quartile had an 85% increase in risk of progressing to CMM (HR: 1.85, 1.12–3.05). Similarly, elevated BRI was associated with higher risks of CMM development in DM and stroke patients (Q4 HR: 1.58, 1.30–1.92 and 1.66, 1.24–2.22, respectively). Additionally, higher BRI levels were linked to an increased mortality risk in DM patients (Q4 HR: 1.18, 1.00–1.39) (Table 2).

### 3.4 Stratified Analyses

In sex‐stratified traditional Cox regression analyses, results demonstrated that compared with Q1, both males and females in the BRI Q4 group exhibited significantly increased HRs for FCMD, CMM, and all‐cause mortality, with females showing more pronounced risk elevations (Table S2). Further sex‐stratified multistate analyses revealed that BRI exerted stronger effects in females than in males, although most transition pathways did not reach statistical significance in females (Table S3).

### 3.5 Sensitivity Analyses

In sensitivity analyses that further adjusted for baseline BMI, BRI remained significantly associated with the risks of FCMD, CMM, and all‐cause mortality. The results of sensitivity analyses excluding participants who experienced events within the first 2 years of follow‐up or excluding those with a history of cancer were not substantially altered. To evaluate the potential impact of participants entering different states on the same date in multistate models, we reanalyzed the data by using four different time intervals and by excluding such participants, and the results of both Cox regression models and the Transition Pattern A multistate model were consistent with our findings (Tables S4–S8).

## 4 Discussion

In this prospective cohort study, we investigated the effect of BRI on the entire progression of CMM. Our findings revealed that elevated BRI was associated with increased risks of FCMD, CMM, and all‐cause mortality. Using multistate models, we observed that BRI significantly affected several transition phases: from healthy to FCMD, healthy to death, FCMD to CMM, and CMM to death. Notably, these effects varied by disease type, with the strongest association for healthy‐to‐DM transitions, while MI patients exhibited the highest progression risk from FCMD to CMM. Additionally, we identified sex‐specific differences, with the impacts of BRI on FCMD, CMM, and all‐cause mortality significantly stronger in females compared to males.

Prior studies have established BRI as a predictor of single CMDs and all‐cause mortality [14, 15, 23]. Our results confirm and extend these findings, showing that elevated BRI significantly raises the risk of both CMDs and all‐cause mortality, and further reveal a robust link between BRI and CMM. Specifically, individuals in the highest BRI quartile had a 199% higher risk of developing CMM than those in the lowest quartile. This result aligns with the cross‐sectional finding of Xiaoru et al., who reported a positive correlation between BRI and CMM prevalence [24].

Some studies have indicated that hypertensive patients with elevated BRI face a significantly increased risk of developing DM and cardiovascular diseases, suggesting a critical role of BRI in the transition from FCMD to CMM [25, 26]. However, these studies focused only on the state of disease and did not evaluate BRI across successive transitions or consider the competing risk of mortality, even though BRI is an established independent risk factor for mortality [23]. To address these deficiencies, we employed a multistate model that accounts for the competing risk of mortality and the effects of different disease transition stages [27]. In Transition Pattern A, participants in the highest BRI quartile showed a 61% greater risk of progressing from FCMD to CMM. This extends the evidence for BRI's pivotal role in CMM development, progression, and prognosis. Notably, a paradoxical trend emerged in the transition from CMM to death: BRI Q4 was associated with a 27% reduced mortality risk. This observation corroborates the “obesity paradox” hypothesis, where elevated BMI or other adiposity metrics may correlate with lower mortality risk in chronic disease populations [28, 29]. One plausible explanation involves metabolic reserve and nutritional buffering: in advanced CMM, excess adiposity may provide energy reserves during catabolic states (e.g., cardiac cachexia), mitigating protein‐energy wasting and preserving organ function [30]. This aligns with clinical evidence demonstrating attenuated mortality in heart failure and coronary artery disease patients with higher BMI or body fat [31, 32]. Our findings support this paradox, suggesting that elevated BRI may correlate with reduced mortality risk in individuals with CMM.

In Transition Pattern B, we conducted an in‐depth analysis of BRI's impact on progression patterns among specific CMDs, including DM, MI, and stroke. The results demonstrated that elevated BRI significantly increased the risk of transitioning from a healthy state to DM, MI, or stroke by 148%, 66%, and 47%, respectively. These findings are consistent with several studies supporting BRI as an independent risk factor for DM and cardiovascular diseases [14, 25]. Interestingly, the association between BRI and both the onset of FCMD and subsequent progression to CMM varied significantly across disease types. Among transitions from a healthy state to specific CMDs, BRI exhibited the strongest association with DM. This phenomenon may stem from the robust causal link between obesity and metabolic dysregulation, which directly drives DM pathogenesis [33, 34]. In contrast, the development of atherosclerosis—a precursor to cardiovascular disease—often requires prolonged exposure to obesity‐related conditions [35]. Furthermore, MI patients with elevated BRI (Q4) faced an 85% higher risk of progressing to CMM compared to the Q1 group, whereas DM and stroke patients showed relatively smaller increases (58% and 66%, respectively). This discovery addresses a critical gap in previous research, highlighting that elevated BRI not only increases MI incidence but also exacerbates its progression to CMM.

Although BMI is widely used to assess obesity, BRI remained independently associated with risks of FCMD, CMM, and all‐cause mortality after adjusting for BMI, suggesting BRI as an independent risk factor apart from BMI. Additionally, we observed that the associations between BRI and risks of FCMD, CMM, and all‐cause mortality were significantly stronger in females than in males. The heightened vulnerability in females may be further amplified by postmenopausal estrogen decline, which attenuates cardioprotective signaling pathways. Estrogen loss reduces endothelial nitric oxide synthase activity, impairs vascular relaxation, and promotes proinflammatory cytokine expression, accelerating cardiometabolic dysfunction [36, 37]. Additionally, estrogen deficiency drives central fat redistribution, potentially enhancing BRI's sensitivity to visceral adiposity in women. However, in female subgroup analysis using the multistate model, only the transition from a healthy state to FCMD exhibited statistically significant associations with BRI, while other transition stages (e.g., from FCMD to CMM, from CMM to death) were not statistically significant. This lack of significance in most transitions may stem from limited statistical power due to the smaller sample size of female participants in our study, potentially obscuring detectable associations.

Although underlying mechanisms linking BRI to CMDs or CMM remain unclear, existing studies suggest that obesity‐driven systemic inflammation and dyslipidemia may serve as shared pathways for the development of both DM and cardiovascular diseases [33, 38]. Specifically, visceral adipose tissue accumulation exacerbates systemic inflammation, which induces insulin resistance and dyslipidemia, thereby promoting the onset of CMDs and progression to CMM [38]. Furthermore, chronic overweight/obesity is associated with hyperactivity of the sympathetic nervous system and renin‐angiotensin‐aldosterone system, which may synergistically accelerate cardiometabolic dysfunction and CMM development [39]. Additionally, lifestyle factors intertwined with obesity—such as sedentary behavior and poor dietary habits—represent modifiable risk drivers for CMDs [40, 41]. Prolonged sedentary work patterns substantially reduce daily energy expenditure, creating a positive energy balance that favors fat storage, particularly visceral adiposity [42]. Sedentary lifestyles also lower basal metabolic rates, compounding metabolic dysregulation and CMD risk. Concurrently, diets rich in calories, saturated fats, and refined sugars not only drive weight gain but also directly impair glucose metabolism and vascular health. Long‐term adherence to such diets independently elevates the risk of CMDs through mechanisms like oxidative stress and endothelial dysfunction [43, 44].

The findings of this study carry significant implications for clinical practice and public health prevention strategies. First, BRI serves as a comprehensive indicator of fat distribution, enabling more precise identification of individuals at high risk for CMDs and CMM, particularly those with normal BMI but central obesity. Consequently, we recommend integrating BRI measurement into routine health screenings and risk assessments as an early detection tool, facilitating timely identification of high‐risk individuals and implementation of personalized interventions. Second, our study is the first to demonstrate BRI's pronounced impact on progression from FCMD to CMM. This underscores the importance of proactively monitoring BRI trajectories in clinical practice to guide early interventions. For instance, clinicians could prioritize BRI‐driven lifestyle adjustments (e.g., reducing sedentary behavior, tailored exercise regimens and dietary plans targeting visceral adiposity) for patients with existing CMDs to mitigate CMM risk. Longitudinal tracking of BRI may serve as a critical biomarker for evaluating intervention efficacy. Critically, future research must establish disease‐specific and sex‐stratified BRI thresholds. Our sex‐stratified analyses revealed stronger associations between elevated BRI and adverse outcomes in females, suggesting that women may benefit from distinct BRI monitoring standards and lower intervention targets. Similarly, cardiometabolic risks vary across CMD subtypes, necessitating disease‐specific BRI cutoffs. Finally, we urge public health policymakers to incorporate CMM into evaluations of BRI‐related disease burden to optimize resource allocation and prevention strategies.

This study has several strengths. First, by employing a multistate model, we were able to account for progression trajectories across disease stages and competing risks simultaneously, yielding less biased estimates compared to traditional Cox proportional hazards regression analyses. Additionally, our data were derived from the Kailuan cohort characterized by a large sample size, strong representativeness, and long‐term follow‐up, with standardized protocols for data collection.

However, some limitations warrant attention. First, while we adjusted for confounders in both multivariable Cox regression and multistate model analyses, covariates (including BRI, lifestyle, and clinical variables) were assessed only at baseline, which may affect the accuracy of risk estimations. Residual confounding (e.g., dietary habits) may also persist. Second, the study population comprised Chinese industrial workers, limiting generalizability to other ethnic or global populations, particularly non‐Asian groups or younger cohorts. External validation is thus required in diverse settings. Third, the results should be interpreted with caution because we could not establish causality; reverse causation might occur due to the observational design. Given these limitations, future studies are needed to corroborate our findings.

## 5 Conclusion

In this prospective cohort study, our findings revealed that elevated BRI differentially influenced the progression from healthy to FCMD, to CMM, and further to death; it also had diverse impacts on disease‐specific transitions. Sex‐specific analyses revealed stronger associations in females. This study highlights dynamic BRI monitoring as a biomarker for early CMM risk identification and intervention prognosis, necessitating disease‐ and sex‐specific prevention strategies. Future research should elucidate BRI's biological mechanisms in CMM pathogenesis.

## Author Contributions

Xi Cai: investigation, methodology, formal analysis, writing – review and editing. Yicheng Liao: investigation, formal analysis, writing – review and editing. Xuemei Yang: investigation, methodology. Yajing Liang: investigation, methodology. Jiajia Ma: formal analysis, writing – review and editing, validation. Ruiyue Liu: investigation. Xinran Wen: methodology. Wenli Yin: resources. Shuohua Chen: writing – review and editing. Guodong Wang: formal analysis. Na Li: methodology. Shouling Wu: resources, supervision. Liufu Cui: conceptualization, resources, supervision, project administration, writing – review and editing.

## Conflicts of Interest

The authors declare no conflicts of interest.

## Supporting information


**Figure S1:** Flow chart of the selection of study participants.
**Figure S2:** Kaplan–Meier incidence rate of FCMD, CMM, and all‐cause mortality according to quartiles of BRI.
**Figure S3:** Multivariable‐adjusted hazard ratios for FCMD, CMM, and all‐cause mortality based on restricted cubic spines.
**Table S1:** Baseline characteristics of the study population according to BRI quartiles.
**Table S2:** Cox regression associations of BRI with FCMD, CMM and all‐cause mortality, stratified by sex.
**Table S3:** The multi‐state model of Transition Pattern A stratified by sex.
**Table S4:** Sensitivity analyses on Cox regression associations of BRI with FCMD, CMM and all‐cause mortality, and the multi‐state model of Transition Pattern A, further adjusted for baseline BMI.
**Table S5:** Sensitivity analyses on Cox regression associations of BRI with FCMD, CMM and all‐cause mortality, and the multi‐state model of Transition Pattern A, excluding events occurring in the first 2 years of follow‐up (*n* = 460).
**Table S6:** Sensitivity analyses on Cox regression associations of BRI with FCMD, CMM and all‐cause mortality, and the multi‐state model of Transition Pattern A, excluding participants with history of cancer (*n* = 5686).
**Table S7:** Sensitivity analyses on Cox regression associations of BRI with FCMD, CMM and all‐cause mortality, and the multi‐state model of Transition Pattern A, by using different time intervals instead of 0.5 day.
**Table S8:** Sensitivity analyses on Cox regression associations of BRI with FCMD, CMM and all‐cause mortality, and the multi‐state model of Transition Pattern A, excluding participants who entered different states on the same date (*n* = 7).

## Data Availability

The data that support the findings of this study are available from the corresponding author upon reasonable request.

## References

[1] E. Di Angelantonio , S. Kaptoge , D. Wormser , et al., “Association of Cardiometabolic Multimorbidity With Mortality,” JAMA 314, no. 1 (2015): 52–60. 26151266 10.1001/jama.2015.7008 PMC4664176

[2] Y. Han , Y. Hu , C. Yu , et al., “Lifestyle, Cardiometabolic Disease, and Multimorbidity in a Prospective Chinese Study,” European Heart Journal 42, no. 34 (2021): 3374–3384. 34333624 10.1093/eurheartj/ehab413 PMC8423468

[3] World Health Organization . Cardiovascular diseases (CVDs) [Internet]. Geneva: World Health Organization [cited 2024 Dec 4]. https://www.who.int/news‐room/fact‐sheets/detail/cardiovascular‐diseases‐(cvds).

[4] GBD 2017 Causes of Death Collaborators , “Global, Regional, and National Age‐Sex‐Specific Mortality for 282 Causes of Death in 195 Countries and Territories, 1980–2017: A Systematic Analysis for the Global Burden of Disease Study 2017,” Lancet 392, no. 10159 (2018): 1736–1788. 30496103 10.1016/S0140-6736(18)32203-7 PMC6227606

[5] S. Jiang , Y. Yang , T. Li , et al., “An Overview of the Mechanisms and Novel Roles of Nrf2 in Cardiovascular Diseases,” Expert Opinion on Therapeutic Targets 20, no. 12 (2016): 1413–1424. 27756179 10.1080/14728222.2016.1250887

[6] T. Li , N. Mu , Y. Yin , L. Yu , and H. Ma , “Targeting AMP‐Activated Protein Kinase in Aging‐Related Cardiovascular Diseases,” Aging and Disease 11, no. 4 (2020): 967–977. 32765957 10.14336/AD.2019.0901 PMC7390518

[7] S. Wang , Y. Fan , X. Feng , et al., “Nicorandil Alleviates Myocardial Injury and Post‐Infarction Cardiac Remodeling by Inhibiting Mst1,” Biochemical and Biophysical Research Communications 495, no. 1 (2018): 292–299. 29127009 10.1016/j.bbrc.2017.11.041

[8] T. Li , S. Jiang , Z. Yang , et al., “Targeting the Energy Guardian AMPK: Another Avenue for Treating Cardiomyopathy?,” Cellular and Molecular Life Sciences 74, no. 8 (2017): 1413–1429. 27815596 10.1007/s00018-016-2407-7 PMC11107559

[9] J. Lu , Y. Wang , L. Hou , Z. Zuo , N. Zhang , and A. Wei , “Multimorbidity Patterns in Old Adults and Their Associated Multi‐Layered Factors: A Cross‐Sectional Study,” BMC Geriatrics 21, no. 1 (2021): 372. 34147073 10.1186/s12877-021-02292-w PMC8214251

[10] K. R. Sahakyan , V. K. Somers , J. P. Rodriguez‐Escudero , et al., “Normal‐Weight Central Obesity: Implications for Total and Cardiovascular Mortality,” Annals of Internal Medicine 163, no. 11 (2015): 827–835. 26551006 10.7326/M14-2525 PMC4995595

[11] D. M. Thomas , C. Bredlau , A. Bosy‐Westphal , et al., “Relationships Between Body Roundness With Body Fat and Visceral Adipose Tissue Emerging From a New Geometrical Model,” Obesity (Silver Spring) 21, no. 11 (2013): 2264–2271. 23519954 10.1002/oby.20408 PMC3692604

[12] N. Zhang , Y. Chang , X. Guo , Y. Chen , N. Ye , and Y. Sun , “A Body Shape Index and Body Roundness Index: Two New Body Indices for Detecting Association Between Obesity and Hyperuricemia in Rural Area of China,” European Journal of Internal Medicine 29 (2016): 32–36. 26895753 10.1016/j.ejim.2016.01.019

[13] S. Rico‐Martín , J. F. Calderón‐García , P. Sánchez‐Rey , C. Franco‐Antonio , M. Martínez Alvarez , and J. F. Sánchez Muñoz‐Torrero , “Effectiveness of Body Roundness Index in Predicting Metabolic Syndrome: A Systematic Review and Meta‐Analysis,” Obesity Reviews 21, no. 7 (2020): e13023. 32267621 10.1111/obr.13023

[14] L. Wu , H. Pu , M. Zhang , H. Hu , and Q. Wan , “Non‐Linear Relationship Between the Body Roundness Index and Incident Type 2 Diabetes in Japan: A Secondary Retrospective Analysis,” Journal of Translational Medicine 20, no. 1 (2022): 110. 35255926 10.1186/s12967-022-03321-x PMC8900386

[15] M. F. H. Maessen , T. M. H. Eijsvogels , R. J. H. M. Verheggen , M. T. E. Hopman , A. L. M. Verbeek , and F. D. Vegt , “Entering a New Era of Body Indices: The Feasibility of a Body Shape Index and Body Roundness Index to Identify Cardiovascular Health Status,” PLoS One 9, no. 9 (2014): e107212. 25229394 10.1371/journal.pone.0107212 PMC4167703

[16] X. Cai , N. Zhao , X. Yang , et al., “The Association Between Body Roundness Index and New‐Onset Hyperuricemia in Chinese Population: The Kailuan Cohort Study,” BMC Public Health 25, no. 1 (2025): 205. 39833792 10.1186/s12889-025-21440-0 PMC11744902

[17] T. Unger , C. Borghi , F. Charchar , et al., “2020 International Society of Hypertension Global Hypertension Practice Guidelines,” Journal of Hypertension 38, no. 6 (2020): 982–1004. 32371787 10.1097/HJH.0000000000002453

[18] X. Kong , Y. Ma , J. Chen , et al., “Evaluation of the Chronic Kidney Disease Epidemiology Collaboration Equation for Estimating Glomerular Filtration Rate in the Chinese Population,” Nephrology, Dialysis, Transplantation 28, no. 3 (2013): 641–651. 10.1093/ndt/gfs491 23197682

[19] W. Jia , J. Weng , D. Zhu , et al., “Standards of Medical Care for Type 2 Diabetes in China 2019,” Diabetes/Metabolism Research and Reviews 35, no. 6 (2019): e3158. 30908791 10.1002/dmrr.3158

[20] K. Thygesen , J. S. Alpert , A. S. Jaffe , et al., “Fourth Universal Definition of Myocardial Infarction (2018),” Circulation 138, no. 20 (2018): e618–e651. 30571511 10.1161/CIR.0000000000000617

[21] “Stroke‐1989. Recommendations on Stroke Prevention, Diagnosis, and Therapy. Report of the WHO Task Force on Stroke and Other Cerebrovascular Disorders,” Stroke 20, no. 10 (1989): 1407–1431. 2799873 10.1161/01.str.20.10.1407

[22] S. Wu , S. An , W. Li , et al., “Association of Trajectory of Cardiovascular Health Score and Incident Cardiovascular Disease,” JAMA Network Open 2, no. 5 (2019): e194758. 31150075 10.1001/jamanetworkopen.2019.4758 PMC6547110

[23] X. Zhang , N. Ma , Q. Lin , et al., “Body Roundness Index and All‐Cause Mortality Among US Adults,” JAMA Network Open 7, no. 6 (2024): e2415051. 38837158 10.1001/jamanetworkopen.2024.15051 PMC11154161

[24] X. Qin , C. Chen , J. Wang , et al., “Association of Adiposity Indices With Cardiometabolic Multimorbidity Among 101,973 Chinese Adults: A Cross‐Sectional Study,” BMC Cardiovascular Disorders 23, no. 1 (2023): 514. 37865773 10.1186/s12872-023-03543-x PMC10590510

[25] X. Cai , S. Song , J. Hu , et al., “Body Roundness Index Improves the Predictive Value of Cardiovascular Disease Risk in Hypertensive Patients With Obstructive Sleep Apnea: A Cohort Study,” Clinical and Experimental Hypertension 45, no. 1 (2023): 2259132. 37805984 10.1080/10641963.2023.2259132

[26] Y. Liu , X. Liu , H. Guan , et al., “Body Roundness Index Is a Superior Obesity Index in Predicting Diabetes Risk Among Hypertensive Patients: A Prospective Cohort Study in China,” Frontiers in Cardiovascular Medicine 8 (2021): 736073. 34869638 10.3389/fcvm.2021.736073 PMC8638826

[27] L. C. de Wreede , M. Fiocco , and H. Putter , “The Mstate Package for Estimation and Prediction in Non‐ and Semi‐Parametric Multi‐State and Competing Risks Models,” Computer Methods and Programs in Biomedicine 99, no. 3 (2010): 261–274. 20227129 10.1016/j.cmpb.2010.01.001

[28] S. Eitmann , P. Matrai , P. Hegyi , et al., “Obesity Paradox in Older Sarcopenic Adults – A Delay in Aging: A Systematic Review and Meta‐Analysis,” Ageing Research Reviews 93 (2024): 102164. 38103840 10.1016/j.arr.2023.102164

[29] Y. Lv , Y. Zhang , X. Li , et al., “Body Mass Index, Waist Circumference, and Mortality in Subjects Older Than 80 Years: A Mendelian Randomization Study,” European Heart Journal 45, no. 24 (2024): 2145–2154. 38626306 10.1093/eurheartj/ehae206 PMC11212828

[30] L. M. Freeman , “The Pathophysiology of Cardiac Cachexia,” Current Opinion in Supportive and Palliative Care 3, no. 4 (2009): 276–281. 19797959 10.1097/SPC.0b013e32833237f1

[31] C. J. Lavie , R. V. Milani , and H. O. Ventura , “Obesity and Cardiovascular Disease: Risk Factor, Paradox, and Impact of Weight Loss,” Journal of the American College of Cardiology 53, no. 21 (2009): 1925–1932. 19460605 10.1016/j.jacc.2008.12.068

[32] A. Romero‐Corral , V. M. Montori , V. K. Somers , et al., “Association of Bodyweight With Total Mortality and With Cardiovascular Events in Coronary Artery Disease: A Systematic Review of Cohort Studies,” Lancet 368, no. 9536 (2006): 666–678. 16920472 10.1016/S0140-6736(06)69251-9

[33] S. Chatterjee , K. Khunti , and M. J. Davies , “Type 2 Diabetes,” Lancet 389, no. 10085 (2017): 2239–2251. 28190580 10.1016/S0140-6736(17)30058-2

[34] J. A. Bell , M. Hamer , G. D. Batty , A. Singh‐Manoux , S. Sabia , and M. Kivimäki , “Incidence of Metabolic Risk Factors Among Healthy Obese Adults: 20‐Year Follow‐Up,” Journal of the American College of Cardiology 66, no. 7 (2015): 871–873. 26271072 10.1016/j.jacc.2015.06.014 PMC4534345

[35] J. P. Reis , C. M. Loria , C. E. Lewis , et al., “Association Between Duration of Overall and Abdominal Obesity Beginning in Young Adulthood and Coronary Artery Calcification in Middle Age,” JAMA 310, no. 3 (2013): 280–288. 23860986 10.1001/jama.2013.7833 PMC4226407

[36] F. Gersh , J. H. O'Keefe , A. Elagizi , C. J. Lavie , and J. A. Laukkanen , “Estrogen and Cardiovascular Disease,” Progress in Cardiovascular Diseases 84 (2024): 60–67. 38272338 10.1016/j.pcad.2024.01.015

[37] A. A. Knowlton and A. R. Lee , “Estrogen and the Cardiovascular System,” Pharmacology & Therapeutics 135, no. 1 (2012): 54–70. 22484805 10.1016/j.pharmthera.2012.03.007 PMC5688223

[38] L. F. Van Gaal , I. L. Mertens , and C. E. De Block , “Mechanisms Linking Obesity With Cardiovascular Disease,” Nature 444, no. 7121 (2006): 875–880. 17167476 10.1038/nature05487

[39] S. B. Heymsfield and T. A. Wadden , “Mechanisms, Pathophysiology, and Management of Obesity,” New England Journal of Medicine 376, no. 15 (2017): 1492. 28402780 10.1056/NEJMc1701944

[40] R. Hariharan , E. N. Odjidja , D. Scott , et al., “The Dietary Inflammatory Index, Obesity, Type 2 Diabetes, and Cardiovascular Risk Factors and Diseases,” Obesity Reviews 23, no. 1 (2022): e13349. 34708499 10.1111/obr.13349

[41] C. J. Lavie , C. Ozemek , S. Carbone , P. T. Katzmarzyk , and S. N. Blair , “Sedentary Behavior, Exercise, and Cardiovascular Health,” Circulation Research 124, no. 5 (2019): 799–815. 30817262 10.1161/CIRCRESAHA.118.312669

[42] F. Lobelo , D. Rohm Young , R. Sallis , et al., “Routine Assessment and Promotion of Physical Activity in Healthcare Settings,” Circulation 137, no. 18 (2018): e495–e522. 29618598 10.1161/CIR.0000000000000559

[43] H. E. Bays , P. R. Taub , E. Epstein , et al., “Ten Things to Know About Ten Cardiovascular Disease Risk Factors,” American Journal of Preventive Cardiology 5 (2021): 100149. 34327491 10.1016/j.ajpc.2021.100149 PMC8315386

[44] S. Wasiak , L. M. Tsujikawa , E. Daze , et al., “Epigenetic BET Reader Inhibitor Apabetalone (RVX‐208) Counters Proinflammatory Aortic Gene Expression in a Diet Induced Obesity Mouse Model and in Human Endothelial Cells,” Atherosclerosis 364 (2023): 10–19. 36455344 10.1016/j.atherosclerosis.2022.11.015

