# Supplementary material for: Body Roundness Index Associated With Cardiometabolic Multimorbidity and Mortality: A Multistate Model
Source: Obesity (Silver Spring). 2025 Sep 28;33(12):2377–86. doi: 10.1002/oby.70032 (PMC12636056; doi:10.1002/oby.70032)
Supplement: Supplementary file 1 — Figure S1: Flow chart of the selection of study participants. Figure S2: Kaplan–Meier incidence rate of FCMD, CMM, and all‐cause mortality according to quartiles of BRI. Figure S3: Multivariable‐adjusted hazard ratios for FCMD, CMM, and all‐cause mortality based on restricted cubic spines. Table S1: Baseline characteristics of the study population according to BRI quartiles. Table S2: Cox regression associations of BRI with FCMD, CMM and all‐cause mortality, stratified by sex. Table S3: The multi‐state model of Transition Pattern A stratified by sex. Table S4: Sensitivity analyses on Cox regression associations of BRI with FCMD, CMM and all‐cause mortality, and the multi‐state model of Transition Pattern A, further adjusted for baseline BMI. Table S5: Sensitivity analyses on Cox regression associations of BRI with FCMD, CMM and all‐cause mortality, and the multi‐state model of Transition Pattern A, excluding events occurring in the first 2 years of follow‐up (n = 460). Table S6: Sensitivity analyses on Cox regression associations of BRI with FCMD, CMM and all‐cause mortality, and the multi‐state model of Transition Pattern A, excluding participants with history of cancer (n = 5686). Table S7: Sensitivity analyses on Cox regression associations of BRI with FCMD, CMM and all‐cause mortality, and the multi‐state model of Transition Pattern A, by using different time intervals instead of 0.5 day. Table S8: Sensitivity analyses on Cox regression associations of BRI with FCMD, CMM and all‐cause mortality, and the multi‐state model of Transition Pattern A, excluding participants who entered different states on the same date (n = 7). [file OBY-33-2377-s001.docx]

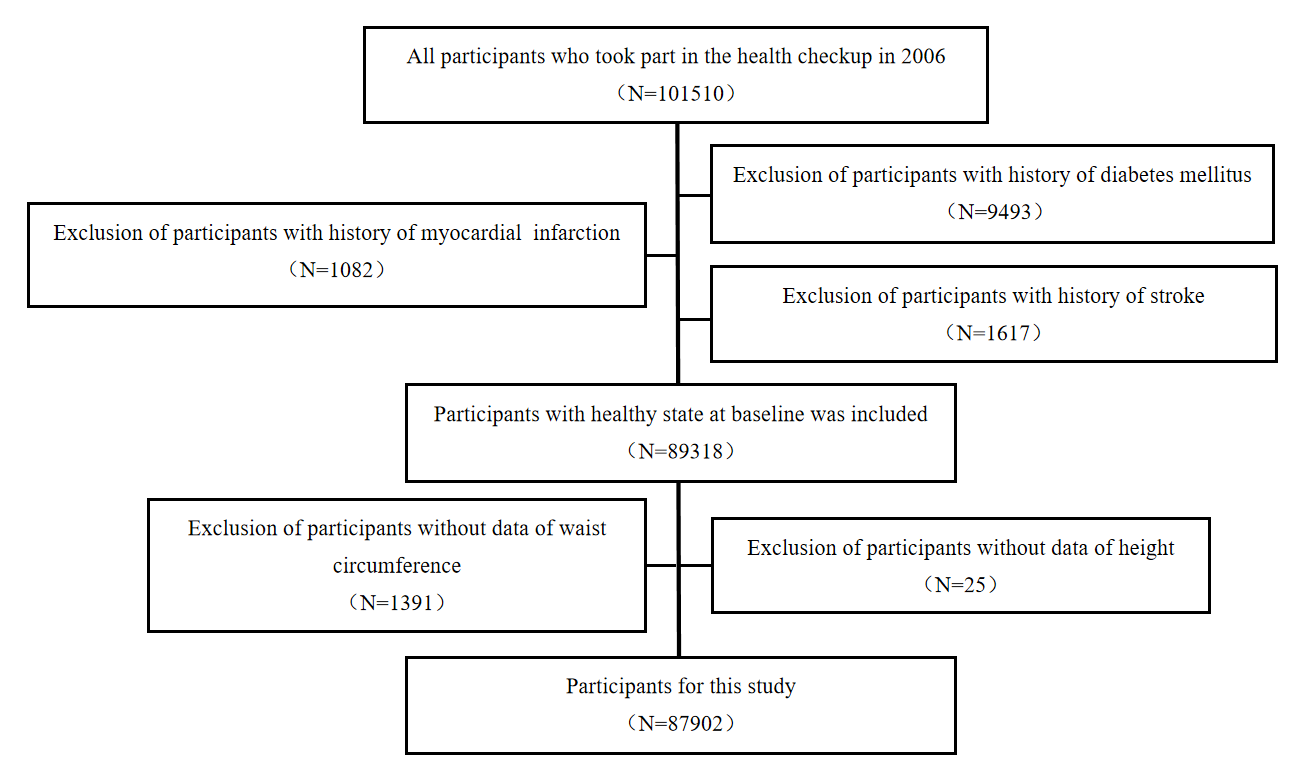


Figure. S1 Flow chart of the selection of study participants.

FCMD CMM All-cause Mortality


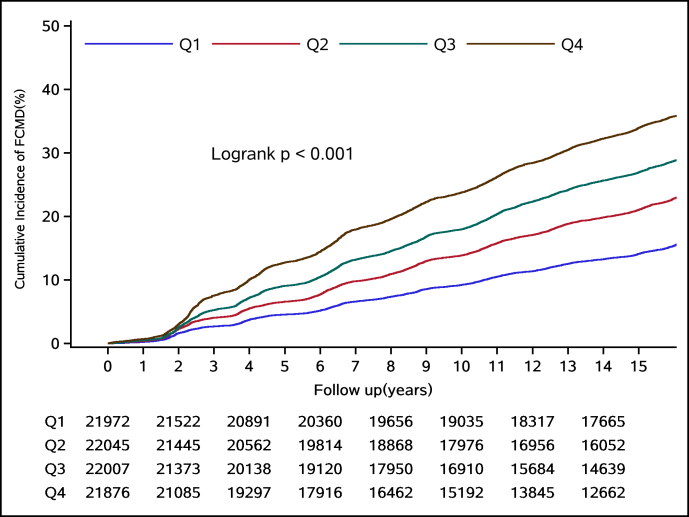

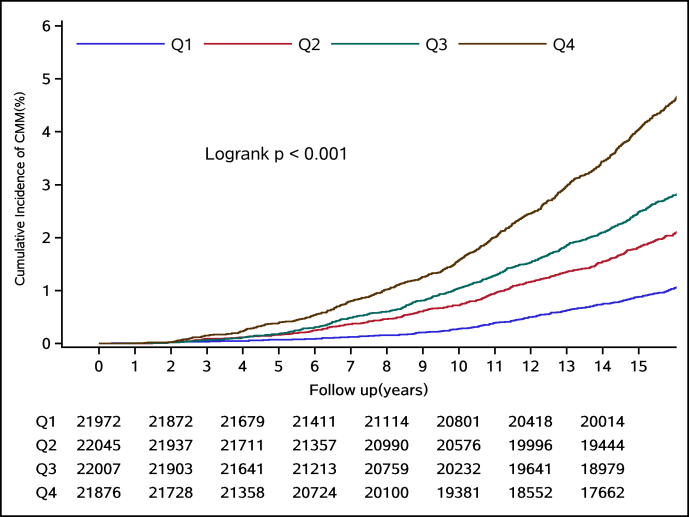

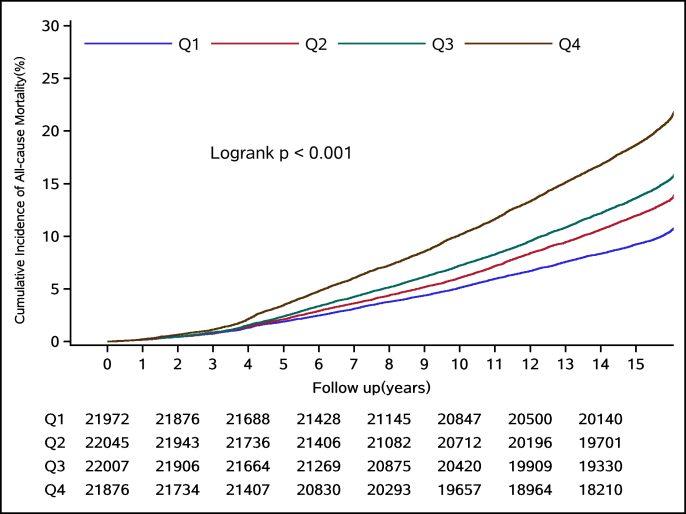


Figure S2 Kaplan-Meier incidence rate of FCMD, CMM, and all-cause mortality according to quartiles of BRI.

Abbreviation: FCMD, first occurrence of cardiometabolic disease; CMM, cardiometabolic multimorbidity.

FCMD CMM All-cause Mortality


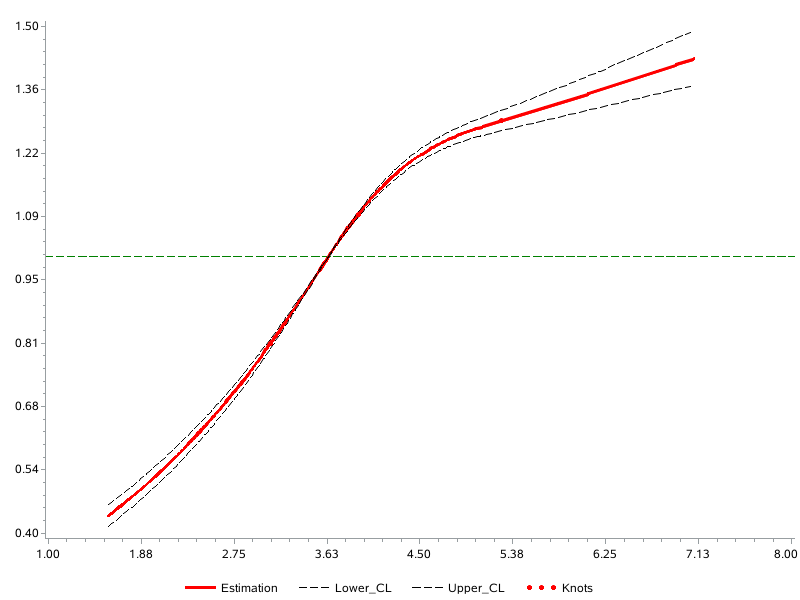

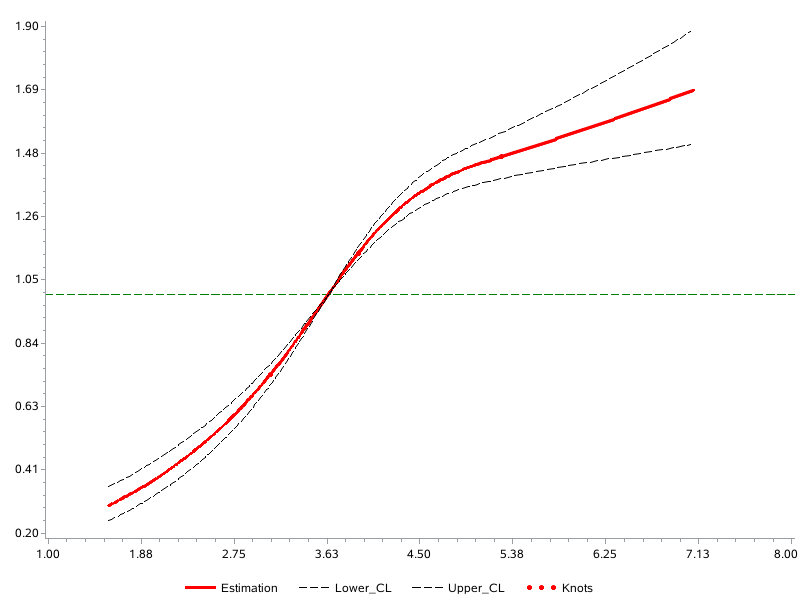

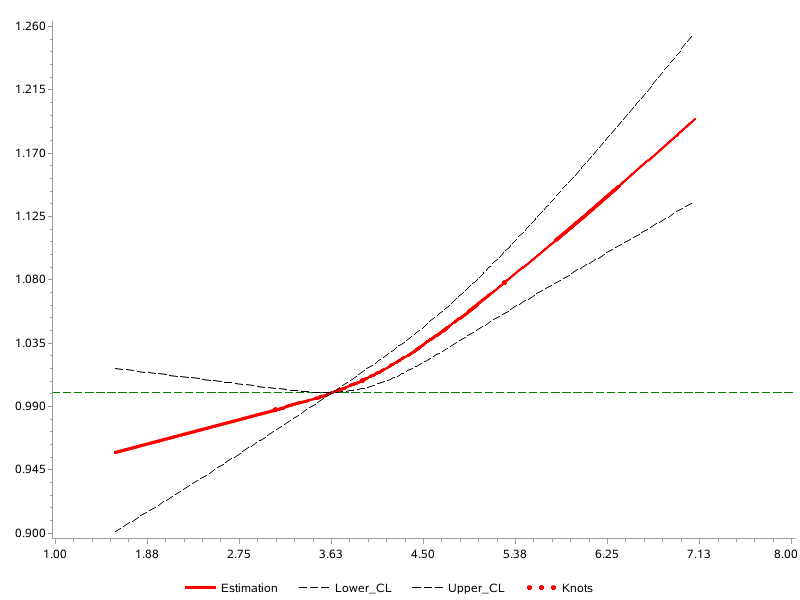


P for overall association＜0.0001

P for nonlinear association=0.0833

P for overall association＜0.0001

P for nonlinear association＜0.0001

P for overall association＜0.0001

P for nonlinear association＜0.0001

Figure S3 Multivariable-adjusted hazard ratios for FCMD, CMM, and all-cause mortality based on restricted cubic spines

with 3 knots at 30th , 60th and 90th percentiles of BRI.

Abbreviation: FCMD, first occurrence of cardiometabolic disease; CMM, cardiometabolic multimorbidity.

Table S1. Baseline characteristics of the study population according to BRI quartiles.

|  | **Overall**  N=87902 | **Quartile 1**  N=21972 | **Quartile 2**  N=22046 | **Quartile 3**  N=22008 | **Quartile 4**  N=21876 | **P value** |
| --- | --- | --- | --- | --- | --- | --- |
| Age (year) | 50.87±12.57 | 46.19±12.96 | 50.05±11.78 | 52.02±11.79 | 55.26±11.95 | ＜0.0001 |
| Male [n (%)] | 69695 (79.30) | 16431 (74.80) | 18068 (82.00) | 18166 (82.50) | 17030 (77.80) | ＜0.0001 |
| LDL-C (mmol/L) | 2.33±0.90 | 2.35±0.81 | 2.39±0.84 | 2.35±0.92 | 2.24±1.02 | ＜0.0001 |
| HDL-C (mmol/L) | 1.55±0.40 | 1.57±0.39 | 1.56±0.40 | 1.54±0.40 | 1.52±0.41 | ＜0.0001 |
| hs-CRP (mg/L) | 0.78 (0.30-2.10) | 0.50 (0.20-1.26) | 0.68 (0.34-1.69) | 0.89 (0.34-2.20) | 1.27 (0.50-3.40) | ＜0.0001 |
| eGFR (ml/min/1.73m^2^) | 83.89±22.54 | 86.84±22.30 | 83.32±23.31 | 82.79±21.64 | 82.59±22.62 | ＜0.0001 |
| WC (cm) | 86.59±10.02 | 75.18±5.33 | 83.57±3.78 | 89.16±4.03 | 98.51±7.43 | ＜0.0001 |
| Height (cm) | 167.45±6.98 | 168.43±6.99 | 168.20±6.71 | 167.29±6.71 | 165.87±7.22 | ＜0.0001 |
| BMI (kg/m^2^) | 24.91±3.47 | 22.09±2.60 | 24.22±2.48 | 25.68±2.59 | 27.67±3.47 | ＜0.0001 |
| FBG (mmol/L) | 5.08±0.69 | 5.02±0.66 | 5.07±0.67 | 5.10±0.71 | 5.14±0.73 | ＜0.0001 |
| Current smoker [n (%)] | 29658 (33.70) | 7865 (35.80) | 7748 (35.10) | 7646 (34.70) | 6399 (29.30) | ＜0.0001 |
| Current drinker [n (%)] | 32560 (37.00) | 8486 (38.60) | 8475 (38.40) | 8459 (38.40) | 7140 (32.60) | ＜0.0001 |
| Physical activity [n (%)] | 77450 (88.10) | 19434 (88.40) | 19547 (88.70) | 19308 (87.70) | 19161 (87.60) | 0.0005 |
| High school and above [n (%)] | 17636 (20.10) | 6376 (29.00) | 4200 (19.10) | 3960 (18.00) | 3100 (14.20) | ＜0.0001 |
| Married [n (%)] | 81290 (92.50) | 20234 (92.10) | 20759 (94.20) | 20428 (92.80) | 19869 (90.80) | ＜0.0001 |

continue supplementary table S1.

|  | **Overall**  N=87902 | **Quartile 1**  N=21972 | **Quartile 2**  N=22046 | **Quartile 3**  N=22008 | **Quartile 4**  N=21876 | **P value** |
| --- | --- | --- | --- | --- | --- | --- |
| Income ≥ 800RMB [n (%)] | 5639 (6.40) | 1591 (7.20) | 1334 (6.10) | 1472 (6.70) | 1242 (5.70) | ＜0.0001 |
| Hypertension [n (%)] | 35958 (40.90) | 5688 (25.90) | 8643 (39.20) | 9950 (45.20) | 11677 (53.40) | ＜0.0001 |
| Antihypertensive drugs [n (%)] | 7853 (8.90) | 836 (3.80) | 1493 (6.80) | 2289 (10.40) | 3235 (14.80) | ＜0.0001 |
| Lipid-lowering drugs [n (%)] | 522 (0.60) | 59 (0.30) | 99 (0.40) | 166 (0.80) | 198 (0.90) | ＜0.0001 |

Note:

Abbreviation: BMI, body mass index; BRI, body roundness index; eGFR, estimated Glomerular filtration rate; FBG, fasting blood glucose; HDL-C, high-density lipoprotein cholesterol; hs-CRP, high-sensitivity C-reactive protein; LDL-C, low-density lipoprotein cholesterol; WC, waist circumference.

Table S2. Cox regression associations of BRI with FCMD, CMM and all-cause mortality, stratified by sex.

|  | **HR (95%CI)** | | | | |
| --- | --- | --- | --- | --- | --- |
|  | **Quartile 1** | **Quartile 2** | **Quartile 3** | **Quartile 4** | **Per SD increase** |
| **Male** |  |  |  |  |  |
| FCMD | Ref. | 1.28 (1.22-1.35) | 1.60 (1.53-1.68) | 1.98 (1.89-2.07) | 1.19 (1.18-1.20) |
| CMM | Ref. | 1.56 (1.31-1.85) | 1.92 (1.63-2.27) | 2.90 (2.47-3.40) | 1.24 (1.21-1.27) |
| Death | Ref. | 1.01 (0.96-1.07) | 1.03 (0.98-1.09) | 1.13 (1.07-1.18) | 1.05 (1.03-1.07) |
| **Female** |  |  |  |  |  |
| FCMD | Ref. | 1.74 (1.54-1.96) | 2.12 (1.88-2.38) | 2.53 (2.25-2.83) | 1.18 (1.15-1.20) |
| CMM | Ref. | 2.37 (1.34-4.19) | 3.50 (2.04-6.01) | 3.95 (2.32-6.75) | 1.19 (1.10-1.29) |
| Death | Ref. | 1.07 (0.87-1.31) | 1.01 (0.83-1.23) | 1.21 (1.01-1.46) | 1.09 (1.04-1.13) |

Note:

Model adjusted for age, sex, smoking status, drinking status, physical activity, income level, education level, marital status, HDL-C, LDL-C, hs-CRP, eGFR, hypertension, anti-hypertensive drugs and lipid-lowering drugs.

Abbreviation: BRI, body roundness index; CMM, cardiometabolic multimorbidity; DM, diabetes mellitus; FCMD, first occurrence of cardiometabolic disease; HR (95% CI), hazard ratio (95% confidence interval); eGFR, estimated Glomerular filtration rate; HDL-C, high-density lipoprotein cholesterol; hs-CRP, high-sensitivity C-reactive protein; LDL-C, low-density lipoprotein cholesterol.

Table S3**.** The multi-state model of transition pattern A stratified by sex.

|  | **No of events (%)** | **HR (95%CI)** | | | | |
| --- | --- | --- | --- | --- | --- | --- |
|  |  | **Quartile 1** | **Quartile 2** | **Quartile 3** | **Quartile 4** | **Per SD increase** |
| **Male** |  |  |  |  |  |  |
| Health → FCMD | 18204 (26.12%) | Ref. | 1.28 (1.22-1.35) | 1.60 (1.53-1.68) | 1.97 (1.89-2.07) | 1.19 (1.17-1.20) |
| Health → Death | 9555 (13.71%) | Ref. | 0.99 (0.93-1.06) | 1.00 (0.94-1.06) | 1.08 (1.02-1.15) | 1.04 (1.02-1.06) |
| FCMD → CMM | 1853 (10.18%) | Ref. | 1.29 (1.09-1.53) | 1.28 (1.09-1.51) | 1.62 (1.38-1.90) | 1.14 (1.09-1.19) |
| FCMD → Death | 3439 (18.89%) | Ref. | 0.98 (0.87-1.11) | 0.96 (0.85-1.07) | 0.99 (0.89-1.11) | 1.01 (0.97-1.04) |
| CMM → Death | 512 (27.63%) | Ref. | 0.78 (0.56-1.10) | 0.72 (0.51-0.99) | 0.70 (0.51-0.96) | 0.89 (0.80-0.98) |
| **Female** |  |  |  |  |  |  |
| Health → FCMD | 3432 (18.84%) | Ref. | 1.74 (1.54-1.96) | 2.12 (1.88-2.38) | 2.53 (2.25-2.84) | 1.18 (1.15-1.20) |
| Health → Death | 865 (4.75%) | Ref. | 1.03 (0.81-1.30) | 1.00 (0.80-1.26) | 1.15 (0.93-1.42) | 1.07 (1.02-1.13) |
| FCMD → CMM | 261 (7.60%) | Ref. | 1.41 (0.80-2.49) | 1.72 (1.01-2.93) | 1.61 (0.95-2.71) | 1.02 (0.92-1.13) |
| FCMD → Death | 359 (10.46%) | Ref. | 0.82 (0.52-1.29) | 0.70 (0.45-1.07) | 0.85 (0.57-1.27) | 1.05 (0.96-1.14) |
| CMM → Death | 52 (19.92%) | Ref. | 2.51 (0.30-2.09) | 0.95 (0.11-7.90) | 1.54 (0.20-12.00) | 0.87 (0.63-1.18) |

Note:

Model adjusted for age, sex, smoking status, drinking status, physical activity, income level, education level, marital status, HDL-C, LDL-C, hs-CRP, eGFR, hypertension, anti-hypertensive drugs and lipid-lowering drugs.

Abbreviation: BRI, body roundness index; CMM, cardiometabolic multimorbidity; DM, diabetes mellitus; FCMD, first occurrence of cardiometabolic disease; HR (95% CI), hazard ratio (95% confidence interval); eGFR, estimated Glomerular filtration rate; HDL-C, high-density lipoprotein cholesterol; hs-CRP, high-sensitivity C-reactive protein; LDL-C, low-density lipoprotein cholesterol.

Table S4. Sensitivity analyses on Cox regression associations of BRI with FCMD, CMM and all-cause mortality, and the multi-state model of transition pattern A, further adjusted for baseline BMI.

|  | **Quartile 1** | **Quartile 2** | **Quartile 3** | **Quartile 4** | **Per SD increase** |
| --- | --- | --- | --- | --- | --- |
| **FCMD** | Ref. | 1.33 (1.27-1.39) | 1.60 (1.53-1.67) | 1.79 (1.70-1.87) | 1.15 (1.13-1.16) |
| **CMM** | Ref. | 1.61 (1.37-1.90) | 1.96 (1.67-2.29) | 2.58 (2.20-3.04) | 1.19 (1.15-1.23) |
| **All-cause Mortality** | Ref. | 1.02 (0.97-1.08) | 1.04 (0.98-1.09) | 1.15 (1.09-1.21) | 1.06 (1.04-1.08) |
| **Transition Pattern A** |  |  |  |  |  |
| Health → FCMD | Ref. | 1.33 (1.27-1.39) | 1.60 (1.53-1.67) | 1.78 (1.70-1.87) | 1.14 (1.13-1.16) |
| Health → Death | Ref. | 1.00 (0.94-1.06) | 1.00 (0.94-1.06) | 1.10 (1.04-1.17) | 1.05 (1.03-1.07) |
| FCMD → CMM | Ref. | 1.30 (1.10-1.53) | 1.32 (1.12-1.54) | 1.59 (1.35-1.87) | 1.11 (1.06-1.16) |
| FCMD → Death | Ref. | 0.98 (0.87-1.10) | 0.95 (0.85-1.06) | 1.02 (0.92-1.14) | 1.03 (0.99-1.07) |
| CMM → Death | Ref. | 0.82 (0.59-1.15) | 0.76 (0.54-1.05) | 0.81 (0.58-1.12) | 0.92 (0.83-1.02) |

Note:

Model adjusted for age, sex, smoking status, drinking status, physical activity, income level, education level, marital status, HDL-C, LDL-C, hs-CRP, eGFR, hypertension, anti-hypertensive drugs and lipid-lowering drugs.

Abbreviation: BRI, body roundness index; CMM, cardiometabolic multimorbidity; DM, diabetes mellitus; FCMD, first occurrence of cardiometabolic disease; HR (95% CI), hazard ratio (95% confidence interval); eGFR, estimated Glomerular filtration rate; HDL-C, high-density lipoprotein cholesterol; hs-CRP, high-sensitivity C-reactive protein; LDL-C, low-density lipoprotein cholesterol.

Table S5. Sensitivity analyses on Cox regression associations of BRI with FCMD, CMM and all-cause mortality, and the multi-state model of transition pattern A, excluding events occurring in the first two years of follow-up (n=460).

|  | **Quartile 1** | **Quartile 2** | **Quartile 3** | **Quartile 4** | **Per SD increase** |
| --- | --- | --- | --- | --- | --- |
| **FCMD** | Ref. | 1.35 (1.29-1.41) | 1.69 (1.61-1.76) | 2.08 (1.99-2.17) | 1.19 (1.18-1.20) |
| **CMM** | Ref. | 1.64 (1.39-1.94) | 2.08 (1.77-2.44) | 3.04 (2.60-3.54) | 1.23 (1.20-1.27) |
| **All-cause Mortality** | Ref. | 1.03 (0.97-1.08) | 1.05 (0.99-1.10) | 1.15 (1.09-1.21) | 1.06 (1.04-1.07) |
| **Transition Pattern A** |  |  |  |  |  |
| Health → FCMD | Ref. | 1.35 (1.29-1.41) | 1.69 (1.61-1.76) | 2.08 (1.99-2.17) | 1.19 (1.18-1.20) |
| Health → Death | Ref. | 1.01 (0.95-1.07) | 1.02 (0.96-1.08) | 1.11 (1.04-1.17) | 1.05 (1.03-1.07) |
| FCMD → CMM | Ref. | 1.30 (1.10-1.54) | 1.33 (1.14-1.56) | 1.63 (1.40-1.90) | 1.12 (1.08-1.17) |
| FCMD → Death | Ref. | 0.97 (0.86-1.09) | 0.93 (0.83-1.04) | 0.98 (0.88-1.09) | 1.01 (0.98-1.04) |
| CMM → Death | Ref. | 0.76 (0.55-1.07) | 0.68 (0.49-0.95) | 0.69 (0.51-0.94) | 0.88 (0.81-0.97) |

Note:

Model adjusted for age, sex, smoking status, drinking status, physical activity, income level, education level, marital status, HDL-C, LDL-C, hs-CRP, eGFR, hypertension, anti-hypertensive drugs and lipid-lowering drugs.

Abbreviation: BRI, body roundness index; CMM, cardiometabolic multimorbidity; DM, diabetes mellitus; FCMD, first occurrence of cardiometabolic disease; HR (95% CI), hazard ratio (95% confidence interval); eGFR, estimated Glomerular filtration rate; HDL-C, high-density lipoprotein cholesterol; hs-CRP, high-sensitivity C-reactive protein; LDL-C, low-density lipoprotein cholesterol.

Table S6. Sensitivity analyses on Cox regression associations of BRI with FCMD, CMM and all-cause mortality, and the multi-state model of transition pattern A, excluding participants with history of cancer (n=5686).

|  | **Quartile 1** | **Quartile 2** | **Quartile 3** | **Quartile 4** | **Per SD increase** |
| --- | --- | --- | --- | --- | --- |
| **FCMD** | Ref. | 1.36 (1.30-1.42) | 1.68 (1.61-1.76) | 2.07 (1.98-2.17) | 1.19 (1.18-1.20) |
| **CMM** | Ref. | 1.58 (1.33-1.86) | 1.98 (1.69-2.33) | 2.91 (2.49-3.40) | 1.23 (1.20-1.26) |
| **All-cause Mortality** | Ref. | 1.02 (0.96-1.09) | 1.06 (1.00-1.12) | 1.19 (1.12-1.26) | 1.07 (1.05-1.09) |
| **Transition Pattern A** |  |  |  |  |  |
| Health → FCMD | Ref. | 1.36 (1.30-1.42) | 1.68 (1.61-1.76) | 2.07 (1.98-2.17) | 1.19 (1.18-1.20) |
| Health → Death | Ref. | 0.99 (0.92-1.06) | 1.03 (0.96-1.10) | 1.15 (1.07-1.23) | 1.06 (1.04-1.08) |
| FCMD → CMM | Ref. | 1.25 (1.06-1.48) | 1.28 (1.09-1.50) | 1.56 (1.34-1.83) | 1.12 (1.07-1.16) |
| FCMD → Death | Ref. | 1.00 (0.88-1.13) | 0.94 (0.83-1.05) | 0.98 (0.87-1.10) | 1.01 (0.97-1.04) |
| CMM → Death | Ref. | 0.79 (0.56-1.11) | 0.70 (0.50-0.98) | 0.72 (0.53-0.99) | 0.90 (0.82-0.98) |

Note:

Model adjusted for age, sex, smoking status, drinking status, physical activity, income level, education level, marital status, HDL-C, LDL-C, hs-CRP, eGFR, hypertension, anti-hypertensive drugs and lipid-lowering drugs.

Abbreviation: BRI, body roundness index; CMM, cardiometabolic multimorbidity; DM, diabetes mellitus; FCMD, first occurrence of cardiometabolic disease; HR (95% CI), hazard ratio (95% confidence interval); eGFR, estimated Glomerular filtration rate; HDL-C, high-density lipoprotein cholesterol; hs-CRP, high-sensitivity C-reactive protein; LDL-C, low-density lipoprotein cholesterol.

Table S7. Sensitivity analyses on Cox regression associations of BRI with FCMD, CMM and all-cause mortality, and the multi-state model of transition pattern A, by using different time intervals instead of 0.5 day.

|  | **Quartile 1** | **Quartile 2** | **Quartile 3** | **Quartile 4** | **Per SD increase** |
| --- | --- | --- | --- | --- | --- |
| **0.5 years** |  |  |  |  |  |
| **FCMD** | Ref. | 1.35 (1.29-1.41) | 1.68 (1.61-1.76) | 2.07 (1.98-2.16) | 1.19 (1.18-1.20) |
| **CMM** | Ref. | 1.63 (1.38-1.92) | 2.05 (1.75-2.40) | 2.99 (2.57-3.49) | 1.23 (1.20-1.26) |
| **All-cause Mortality** | Ref. | 1.02 (0.97-1.07) | 1.04 (0.98-1.09) | 1.14 (1.09-1.20) | 1.06 (1.04-1.07) |
| **Transition Pattern A** |  |  |  |  |  |
| Health → FCMD | Ref. | 1.35 (1.29-1.41) | 1.68 (1.61-1.76) | 2.07 (1.98-2.16) | 1.19 (1.18-1.20) |
| Health → Death | Ref. | 1.00 (0.94-1.06) | 1.00 (0.94-1.06) | 1.10 (1.03-1.16) | 1.04 (1.03-1.06) |
| FCMD → CMM | Ref. | 1.30 (1.10-1.53) | 1.32 (1.13-1.55) | 1.61 (1.38-1.88) | 1.12 (1.08-1.16) |
| FCMD → Death | Ref. | 0.98 (0.87-1.10) | 0.94 (0.84-1.05) | 0.99 (0.89-1.10) | 1.01 (0.98-1.05) |
| CMM → Death | Ref. | 0.82 (0.59-1.14) | 0.73 (0.53-1.01) | 0.73 (0.54-0.99) | 0.89 (0.81-0.97) |
| **1 year** |  |  |  |  |  |
| **FCMD** | Ref. | 1.35 (1.29-1.41) | 1.68 (1.61-1.76) | 2.07 (1.98-2.16) | 1.19 (1.18-1.20) |
| **CMM** | Ref. | 1.63 (1.38-1.92) | 2.05 (1.75-2.40) | 2.99 (2.57-3.49) | 1.23 (1.20-1.26) |
| **All-cause Mortality** | Ref. | 1.02 (0.97-1.07) | 1.04 (0.98-1.09) | 1.14 (1.09-1.20) | 1.06 (1.04-1.07) |
| **Transition Pattern A** |  |  |  |  |  |
| Health → FCMD | Ref. | 1.35 (1.29-1.41) | 1.68 (1.61-1.76) | 2.07 (1.98-2.16) | 1.19 (1.18-1.20) |
| Health → Death | Ref. | 1.00 (0.94-1.06) | 1.00 (0.94-1.06) | 1.10 (1.03-1.16) | 1.04 (1.03-1.06) |
| FCMD → CMM | Ref. | 1.30 (1.10-1.53) | 1.32 (1.13-1.55) | 1.61 (1.38-1.87) | 1.12 (1.08-1.16) |

Continue supplementary table S7.

|  | **Quartile 1** | **Quartile 2** | **Quartile 3** | **Quartile 4** | **Per SD increase** |
| --- | --- | --- | --- | --- | --- |
| FCMD → Death | Ref. | 0.98 (0.87-1.10) | 0.94 (0.84-1.05) | 0.99 (0.89-1.10) | 1.01 (0.98-1.05) |
| CMM → Death | Ref. | 0.82 (0.59-1.14) | 0.73 (0.52-1.00) | 0.73 (0.53-0.99) | 0.89 (0.81-0.97) |
| **3 years** |  |  |  |  |  |
| **FCMD** | Ref. | 1.35 (1.29-1.41) | 1.68 (1.61-1.76) | 2.07 (1.98-2.16) | 1.19 (1.18-1.20) |
| **CMM** | Ref. | 1.63 (1.38-1.92) | 2.05 (1.75-2.40) | 2.99 (2.57-3.49) | 1.23 (1.20-1.26) |
| **All-cause Mortality** | Ref. | 1.02 (0.97-1.07) | 1.04 (0.98-1.09) | 1.14 (1.09-1.20) | 1.06 (1.04-1.07) |
| **Transition Pattern A** |  |  |  |  |  |
| Health → FCMD | Ref. | 1.35 (1.29-1.41) | 1.68 (1.61-1.76) | 2.07 (1.98-2.16) | 1.19 (1.18-1.20) |
| Health → Death | Ref. | 1.00 (0.94-1.06) | 1.00 (0.94-1.06) | 1.10 (1.03-1.16) | 1.04 (1.03-1.06) |
| FCMD → CMM | Ref. | 1.30 (1.10-1.53) | 1.32 (1.13-1.55) | 1.61 (1.38-1.87) | 1.12 (1.08-1.16) |
| FCMD → Death | Ref. | 0.98 (0.87-1.10) | 0.94 (0.84-1.05) | 0.99 (0.89-1.10) | 1.01 (0.98-1.05) |
| CMM → Death | Ref. | 0.82 (0.59-1.14) | 0.73 (0.52-1.01) | 0.73 (0.53-0.99) | 0.89 (0.81-0.97) |
| **5 years** |  |  |  |  |  |
| **FCMD** | Ref. | 1.35 (1.29-1.41) | 1.68 (1.61-1.76) | 2.07 (1.98-2.16) | 1.19 (1.18-1.20) |
| **CMM** | Ref. | 1.63 (1.38-1.92) | 2.05 (1.75-2.40) | 2.99 (2.57-3.49) | 1.23 (1.20-1.26) |
| **All-cause Mortality** | Ref. | 1.02 (0.97-1.07) | 1.04 (0.98-1.09) | 1.14 (1.09-1.20) | 1.06 (1.04-1.07) |
| **Transition Pattern A** |  |  |  |  |  |
| Health → FCMD | Ref. | 1.35 (1.29-1.41) | 1.68 (1.61-1.76) | 2.07 (1.98-2.16) | 1.19 (1.18-1.20) |
| Health → Death | Ref. | 1.00 (0.94-1.06) | 1.00 (0.94-1.06) | 1.10 (1.03-1.16) | 1.04 (1.03-1.06) |

Continue supplementary table S7.

|  | **Quartile 1** | **Quartile 2** | **Quartile 3** | **Quartile 4** | **Per SD increase** |
| --- | --- | --- | --- | --- | --- |
| FCMD → CMM | Ref. | 1.30 (1.10-1.53) | 1.32 (1.13-1.55) | 1.61 (1.38-1.88) | 1.12 (1.08-1.16) |
| FCMD → Death | Ref. | 0.98 (0.87-1.10) | 0.94 (0.84-1.05) | 0.99 (0.89-1.10) | 1.01 (0.98-1.05) |
| CMM → Death | Ref. | 0.82 (0.59-1.14) | 0.73 (0.52-1.01) | 0.73 (0.53-0.99) | 0.89 (0.81-0.97) |

Note:

Model adjusted for age, sex, smoking status, drinking status, physical activity, income level, education level, marital status, HDL-C, LDL-C, hs-CRP, eGFR, hypertension, anti-hypertensive drugs and lipid-lowering drugs.

Abbreviation: BRI, body roundness index; CMM, cardiometabolic multimorbidity; DM, diabetes mellitus; FCMD, first occurrence of cardiometabolic disease; HR (95% CI), hazard ratio (95% confidence interval); eGFR, estimated Glomerular filtration rate; HDL-C, high-density lipoprotein cholesterol; hs-CRP, high-sensitivity C-reactive protein; LDL-C, low-density lipoprotein cholesterol.

Table S8. Sensitivity analyses on Cox regression associations of BRI with FCMD, CMM and all-cause mortality, and the multi-state model of transition pattern A, excluding participants who entered different states on the same date (n=7).

|  | **Quartile 1** | **Quartile 2** | **Quartile 3** | **Quartile 4** | **Per SD increase** |
| --- | --- | --- | --- | --- | --- |
| **FCMD** | Ref. | 1.35 (1.29-1.41) | 1.69 (1.62-1.76) | 2.08 (2.00-2.17) | 1.19 (1.18-1.20) |
| **CMM** | Ref. | 1.64 (1.39-1.94) | 2.07 (1.76-2.42) | 3.02 (2.59-3.53) | 1.23 (1.20-1.26) |
| **All-cause Mortality** | Ref. | 1.02 (0.97-1.07) | 1.04 (0.98-1.09) | 1.14 (1.09-1.20) | 1.06 (1.04-1.07) |
| **Transition Pattern A** |  |  |  |  |  |
| Health → FCMD | Ref. | 1.35 (1.29-1.41) | 1.69 (1.61-1.76) | 2.08 (1.99-2.17) | 1.19 (1.18-1.20) |
| Health → Death | Ref. | 1.00 (0.94-1.06) | 1.00 (0.94-1.06) | 1.10 (1.03-1.16) | 1.04 (1.03-1.06) |
| FCMD → CMM | Ref. | 1.30 (1.11-1.54) | 1.33 (1.14-1.56) | 1.63 (1.39-1.88) | 1.12 (1.08-1.17) |
| FCMD → Death | Ref. | 0.98 (0.87-1.10) | 0.94 (0.84-1.05) | 0.99 (0.89-1.10) | 1.01 (0.98-1.05) |
| CMM → Death | Ref. | 0.82 (0.59-1.15) | 0.73 (0.53-1.02) | 0.74 (0.54-1.00) | 0.89 (0.81-0.98) |

Note:

Model adjusted for age, sex, smoking status, drinking status, physical activity, income level, education level, marital status, HDL-C, LDL-C, hs-CRP, eGFR, hypertension, anti-hypertensive drugs and lipid-lowering drugs.

Abbreviation: BRI, body roundness index; CMM, cardiometabolic multimorbidity; DM, diabetes mellitus; FCMD, first occurrence of cardiometabolic disease; HR (95% CI), hazard ratio (95% confidence interval); eGFR, estimated Glomerular filtration rate; HDL-C, high-density lipoprotein cholesterol; hs-CRP, high-sensitivity C-reactive protein; LDL-C, low-density lipoprotein cholesterol.
